# A missense mutation in the *C. elegans src-2* tyrosine-protein kinase reduces brood size and enhances embryonic morphogenesis defects in *src-1(RNAi)* conditions

**DOI:** 10.17912/micropub.biology.000872

**Published:** 2023-07-11

**Authors:** Xiaofei Bai, Rebecca Green, Tao Cai, Karen Oegema, Andy Golden

**Affiliations:** 1 Biology, University of Florida, Gainesville, Florida, United States; 2 Genetics Institute, University of Florida, Gainesville, Florida, United States; 3 Laboratory of Biochemistry and Genetics, National Institute of Diabetes and Digestive and Kidney Diseases, National Institutes of Health, Bethesda, MD; 4 Department of Cell and Developmental Biology, School of Biological Sciences, University of California, San Diego, La Jolla, CA; 5 Ludwig Institute for Cancer Research, San Diego, CA; 6 Department of Cellular and Molecular Medicine, University of California, San Diego, La Jolla, CA

## Abstract

Goldenhar Syndrome is a rare congenital disorder characterized by hemifacial microsomia. Although select mutations have been mapped for this disorder, the genetic etiologies in the majority of cases remain unknown. A recent clinical report of a Goldenhar Syndrome patient identified a homozygous missense mutation in *FRK* , a gene associated with various types of cancer. In this work, we precisely modeled the disease-associated missense mutation in the *C. elegans* FRK ortholog *src-2 ,* using CRISPR/Cas9 gene editing, and investigated the physiological role of this mutation and the *src-2* gene. In addition, we generated a conserved variant in *src-1* ( *FYN* ortholog) to assess the functional redundancy of the conserved variant. The putative pathogenic variants *src-1 (Val190Ile)* or *src-2 (Val170Ile)* caused only subtle phenotypes, suggesting that these mutations alone are not sufficient to explain the facial deformities observed in the Goldenhar Syndrome patient. However, the *src-2 (Val170Ile)* mutant exhibited reduced brood size and moderately enhanced embryonic developmental phenotypes, including epidermal and neuronal patterning defects, in the *src-1 (RNAi)* condition, indicating that the *src-2 (Val170Ile)* locus could play a supportive role during developmental processes. Overall, however, these studies showed that *src-1 /FYN* is essential for regulating embryogenesis and morphogenesis, while *src-2 /FRK* is largely dispensable for normal embryonic development, suggesting *FYN* , not *FRK* , is the dominant non-receptor protein kinase during embryonic development in *C. elegans* .

**Figure 1. src-2 (Val170Ile ) mutant exhibits reduced brood size and enhances embryonic morphogenesis defects in src-1 (RNAi) conditions:**
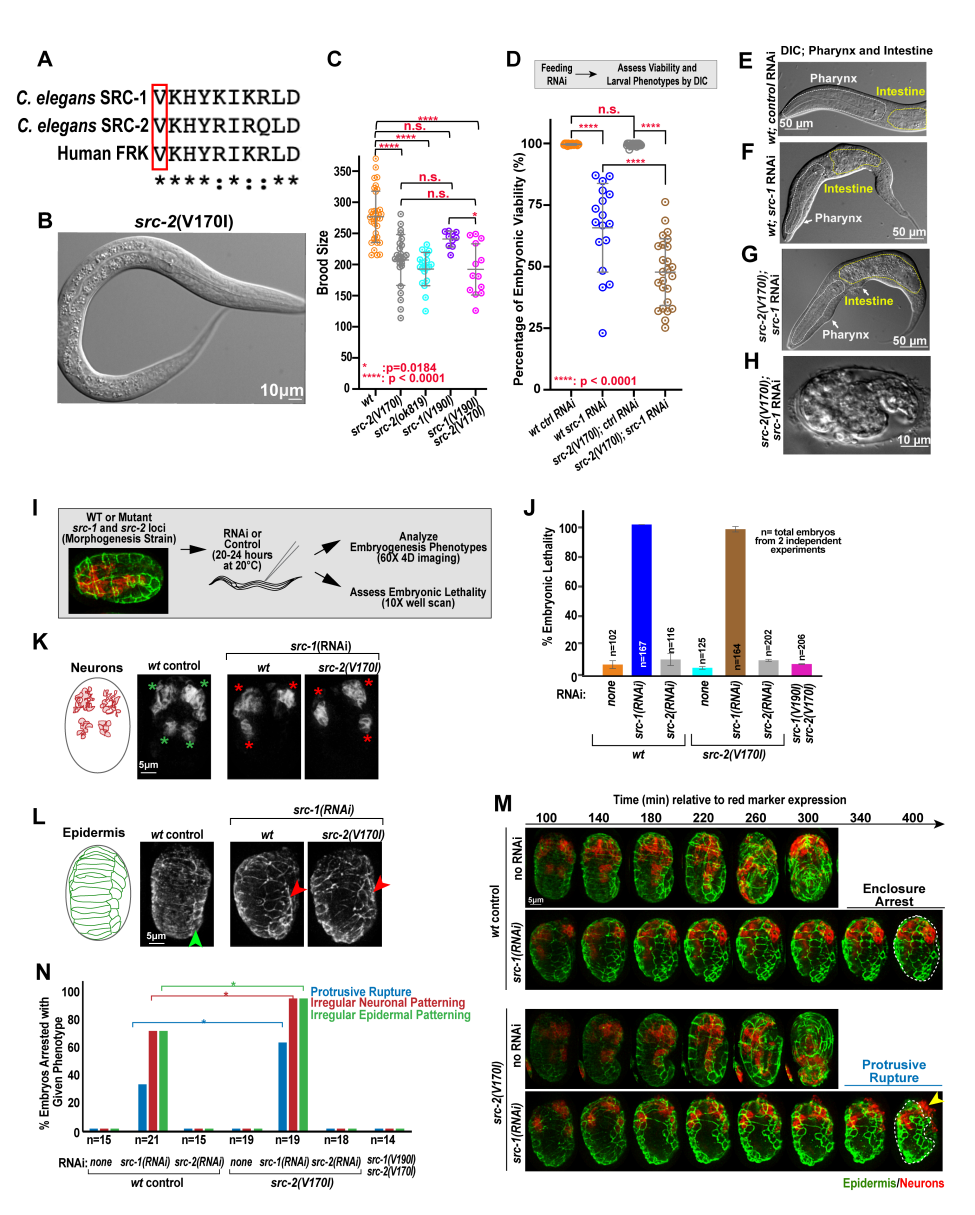
(A) Sequence alignment of the conserved valine residue of *C. elegans* SRC-1 , SRC-2 , and human FRK. (B) Representative DIC images of *src-2 (Val170Ile)* mutant. (C) Quantification of brood size of wild type and missense mutants of *src-1* and *src-2* . (D) Percent embryonic viability after feeding control or *src-1 (RNAi)* in wild-type, and missense mutants of *src-1* and *src-2* . (E-H) Representative DIC images of wild type and defective larvae and embryos in missense mutants or following *src-1 (RNAi)* . (I) Schematic outlining the 4D embryonic developmental imaging regime. (J) Plot highlights embryonic lethality data captured by 10x whole well-scan, following overnight imaging regime. (K) Schematic and images show initial patterning of neuronal precursors in wild-type control embryos and after *src-1 (RNAi)* in control and *src-2 (Val170Ile)* mutant embryos. Green asterisks highlight patterning of neurons into four quadrants (expected), and red asterisks highlight abnormal neuronal number and/or position. (L) Images show epidermal patterning from the dorsal surface in wild-type control embryos, and after *src-1 (RNAi* ) in control and *src-2 (Val170Ile)* mutant embryos. Green arrow points to uniform row of seam cells along the lateral surface in control embryo, whereas red arrow indicates disorganized population of seam cells. (M) Images depict time series during enclosure and elongation (controls) and arrest at enclosure or protrusive rupture (yellow arrow) at enclosure in control or *src-1 (RNAi)* embryos (respectively). Epidermis shown in green, and neurons shown in red. Time is relative to initial red marker expression. (N) Graph quantifies the relative frequency of embryonic phenotypes observed in all tested conditions. Asterisks indicate P<0.05 by one tailed chi-square test in (M). P-values: * = 0.0184 (C); **** <0.0001 (C-D) (one-way ANOVA test). Asterisks indicate P<0.05 by one tailed chi-square test (N). Scale bars are indicated in each graphic.

## Description

Fyn-related Src family tyrosine kinase (FRK) belongs to the family of non-receptor protein kinases, serving as a signaling intermediary during G1 and S phase to negatively regulate cell proliferation (Hua et al., 2014; Lee et al., 1994). Dysfunction of *FRK* gene has been linked to various types of cancers, including breast and brain cancers (Goel and Lukong, 2016). In a recent clinical report, a homozygous missense mutation of the *FRK* gene, NM_002031.3 (FRK): c.484G>A (p.Val162Ile) ( https://www.ncbi.nlm.nih.gov/clinvar/variation/VCV001162776.2 ), was identified in a Goldenhar Syndrome patient presenting with hemifacial microsomia (HFM). HFM is a common birth defect and although several chromosomal abnormalities and causal gene variants have been identified, genetic etiologies in the majority of cases remain unknown. The phenotypic features associated with this disease suggest that defects may arise from abnormities in cranial neural crest cells (CNCC) (Wang et al., 2020), which are an ectoderm-derived set of cells that migrate to the pharyngeal arches and facilitate craniofacial development (Bhatt et al., 2013; Siismets and Hatch, 2020). To investigate the physiological effect of this putative pathogenic variant, we precisely modeled the Val170Ile variant in the *C. elegans* *FRK* ortholog *src-2* , using CRISPR/Cas9 gene editing. Since a sequence alignment indicated that *src-1 (FYN* ortholog) is the paralog of *src-2* in *C. elegans* , we also generated the conserved variant Val190Ile in *src-1* to characterize the functional redundancy of the variant ( **Fig. 1A** ).

Homozygous animals carrying *src-1 (Val190Ile)* mutant alone, *src-2 (Val170Ile* ) mutant alone, and *src-1 (Val190Ile); src-2 (Val170Ile)* double mutant fail to exhibit obvious morphological defects in hatched animals ( **Fig. 1B** ). However, animals carrying the *src-2 (Val170Ile)* mutation have reduced brood sizes, similar to those of a null *src-2* mutant, *src-2 ( ok819 )* , which bears a full-length deletion of the *src-2* gene ( **Fig. 1C** ). The *src-1 (Val190Ile)* variant alone does not cause a significant reduction of brood size compared to wild type ( **Fig. 1C** ). Additionally, the *src-1 (Val190Ile) src-2 (Val170Ile)* double mutant shows a similar brood size when compared to the *src-2 (Val170Ile)* mutant alone, suggesting that the *src-1 (Val190Ile)* variant does not have functional redundancy with the *src-2 (Val170Ile)* variant ( **Fig. 1C** ) with respect to its role regulating brood size.

While the *src-2 (Val170Ile* ) mutant alone does not result in morphological or embryonic lethality defects ( **Fig. 1B** ), depletion of *src-1 ,* by feeding RNAi, in either wildtype or *src-2 (Val170Ile)* is associated with a variety of tissue morphogenesis defects, including abnormal pharyngeal, intestinal, and epidermal morphogenesis ( **Fig. 1D -H** ); larval morphogenesis defects and embryonic lethality phenotypes are enhanced when *src-1 (RNAi)* is combined with *src-2 (Val170Ile* ) mutant condition ( **Fig. 1D -H** ). These findings are consistent with prior work suggesting *src-1* is the predominant FRK/SRC during embryonic development in *C. elegans* ; lethality studies have shown essential roles for *src-1* , but not *src-2* during embryogenesis (Bei et al., 2002; Hirose et al., 2003) and single-cell sequencing data has shown that *src-1* is widely expressed across all tissues during development, while *src-2* expression is restricted to developing pharynx and adjacent regions (Packer et al., 2019).

To further assess the nature of the embryonic defects when *FRK* gene expression is disrupted, we employed a previously described automated 4D imaging regime, which enables collection of semi-high throughput embryogenesis data and allows for profiling of embryonic developmental phenotypes following perturbation (Wang et al., 2019) ( Fig. 1I -N). For this experiment, we used a morphogenesis reporter strain (Wang et al., 2019) that reports specifically on ectodermally derived tissue, since the HFM defect in humans likely arises from mis-patterning of this tissue during development. Specifically, this strain expresses a GFP-tagged epithelial junction marker in the epidermis (P *dlg-1 Δ7* :: DLG-1 ::GFP) and a mCherry-tagged plasma membrane marker in a subset of the developing neurons (P *cnd-1* ::mCherry::PH). In this strain, or in the same strain background carrying the *src-2 (Val170Ile)* or *src-1 (Val190Ile)* ; *src-2 (Val170Ile)* double mutation, created by CRISPR gene editing, we depleted *src-1* and *src-2* by injection RNAi ( **Fig. 1J -N** ). Consistent with our previous results, the *src-2 (Val170Ile)* mutant alone and the *src-1 (V190I)* *src-2 (Val170Ile)* double mutant embryos do not exhibit significant defects or embryonic lethality in this strain background ( **Fig. 1J** ), suggesting that the patient isolated mutation is not sufficient to trigger developmental defects in *C. elegans* . Next, we performed *src-1 (RNAi)* , by injection, in wild type or mutant *src-2 (Val170Ile)* conditions to examine if these mutations can enhance *src-1 (RNAi)* phenotypes. We found that *src-1 (RNAi)* alone or in combination with the *src-2 (Val170Ile)* mutation results in penetrant embryonic lethality, whereas control and *src-2 (RNAi)* depletion conditions do not ( **Fig 1J** ).

To understand the nature of the embryonic lethality in *src-1 (RNAi)* and determine if enhancement is observed when the *src-2* missense mutation is combined with this condition, we analyzed the high-resolution 4D embryonic development data; this revealed that most embryos arrest at or near comma stage in *src-1 (RNAi)* depleted embryos in control or the *src-2 (Val170Ile)* strain (62% and 74% respectively) ( **Fig. 1N** ). We did not observe gross abnormalities in the proliferation of cells, prior to arrest, in the morphogenesis reporter strain in any background. Similarly, the same perturbation conditions were tested using a germ-layer reporter strain, which marks endoderm, ectoderm, and mesoderm cells (Wang et al., 2019). We found the nuclear counts for each germ layer at the comma stage to be largely comparable to controls. However, despite largely normal nuclear counts, the arrested embryos under *src-1 (RNAi)* conditions frequently exhibit enclosure defects, disrupted germ-layer organization, and irregular neuronal and hypodermal patterning ( **Fig. 1K -N** ), suggesting that tissue mis-patterning may underpin the observed enclosure defects. While both *src-1 (RNAi)* in control embryos and in the *src-2 (Val170Ile)* strain tended to result in defects during epidermal enclosure, *src-1 (RNAi)* in combination with *src-2 (Val170Ile)* was more likely to result in embryos that undergo a dramatic protrusive rupture event, whereby the enclosing epidermal tissue retracts and releases or partially releases neuronal tissue (33% vs 63% respectively) ( **Fig. 1M, N** ). A similar uptick in prevalence was observed in irregular neuronal and epidermal patterning in *src-1 (RNAi)* in the *src-2 (Val170Ile)* strain, relative to *src-1 (RNAi)* alone ( **Fig. 1K** ). During development, neuronal precursors are established in four quadrants in control embryos, with precise symmetry along the anterior-posterior axis. However, in *src-1 (RNAi)* control embryos and in the *src-1 (RNAi)* , *src-2 (Val170Ile)* strain condition, abnormal patterning phenotypes were observed in 71% and 95% of the imaged embryos (respectively); both conditions frequently exhibit three asymmetrical loci instead of the expected 4 symmetrical loci (compare red to green asterisks, **Fig. 1K, N** ). Similarly, epidermal patterning in control embryos is characterized by an organized strip of dorsal hypodermal cells and a single row of seam cells (arrows) that stretch along each of the lateral surfaces during enclosure. In contrast, in *src-1 (RNAi)* and in the *src-1 (RNAi)* , *src-2 (Val170Ile)* condition, a disorganized epidermis is observed, with ill-defined dorsal hypodermis and seam cells (71% and 95%, **Fig. 1L, N** ). The co-occurrence of neuronal and epidermal asymmetrical patterning defects in these conditions may indicate a general disruption of ectoderm-derived tissues or may suggest a feedback mechanism between neuronal and epidermal tissue morphogenic processes in the *src-1 (RNAi)* condition. Altogether, developmental phenotypes, including severe neuronal patterning, epidermal patterning, and protrusive rupture phenotypes were enhanced in the *src-1 (RNAi)* *src-2 (Val170Ile)* combined condition compared to *src-1 (RNAi)* controls alone by 24%, 24%, and 30%, respectively (P= 0.0263, P= 0.0263, and P= 0.0296 by one-tailed chi-square test) ( **Fig. 1N** ). The *src-2 (Val170Ile)* allele modestly enhances the *src-1 (RNAi)* phenotype suggesting that SRC-2 has a role in supporting the function of its paralog SRC-1 during embryonic development. Alternatively, we cannot rule out the possibility that the brood size effects in *src-2 (Val170Ile)* may synergize with *src-1 (RNAi)* in unexpected ways to manifest in developmental defects, due to altered loading kinetics of the injected RNA into forming embryos.

Our studies indicate that *src-1 /FYN* is an essential gene in regulating embryogenesis and morphogenesis, whereas *src-2 /FRK* is largely dispensable for normal embryonic development, consistent with prior work (Bei et al., 2002) . This suggests that FYN, not FRK, is the dominant non-receptor protein kinase during development in *C. elegans* . We find that the developmental defects observed in the *src-1 (RNAi)* condition are largely due to asymmetric mis-patterning of ectodermal tissues, including both the epidermis and neurons. The putative pathogenic variants *src-1 (Val190Ile)* or *src-2 (Val170Ile)* caused only subtle phenotypes, which suggests that these mutations are not sufficient to explain the facial deformities observed in the Goldenhar Syndrome patient. However, we cannot rule out the possibility that the human *FYN/FRK* could have acquired new molecular interactions that are only required in human facial tissues that are specifically disrupted by the mutation. Nevertheless, the *src-2 (Val170Ile)* mutant exhibits reduced brood size and moderately enhanced embryonic developmental phenotypes in the *src-1 (RNAi)* condition. Thus, we cannot rule out that the *src-2 (Val170Ile)* locus could play a supportive role during developmental processes.

## Methods

4D imaging was conducted as previously described (Wang, Ochoa, Khaliullin 2019). Briefly, L4 stage animals (strains used OD1719 , OD1689 , AG605 , AG606 , and AG607 ) were either not injected or were injected with dsRNA, manufactured in house, directed at *src-1* (oligos: TAATACGACTCACTATAGGGCAATGTGATCATCCGAATC, AATTAACCCTCACTAAAGGGATACGGAACCTGTCCCTTT) or *src-2* (oligos: TAATACGACTCACTATAGGCGCTGTGAAAAAGCTAAAGG, AATTAACCCTCACTAAAGGTGAGCAGGATTCCAAAACTC) 20-22 hours prior to imaging. The injected dsRNA were used in the panel (J-M). Embryos were dissected from gravid adults in ice cold M9 + 0.1mg/ml tetramisole hydrochloride and transferred to 384-well glass bottom plate, which was kept on ice until wells were loaded and ready to be imaged. Embryos were imaged at 18°C by 2 micron steps, every 20 minutes for approximately 10 hours on a CV1000 confocal scanner box, equipped with a microlens-enhanced dual Nipkow spinning disk (Yokogawa Electric Corporation) and a 60×1.35NA U-PlanApo objective. Imaging was performed in a temperature-controlled room and data collection was done using CellVoyager software (Yokogawa Electric Corporation). Following overnight imaging, a whole well brightfield scan was performed at 10× to capture embryonic lethality and larval phenotypes. For more details on image acquisition see Wang, Ochoa, Khaliullin 2019. Individual embryos were cropped from the broader field using a custom embryo cropping algorithm (10, 11) and developmental arrest points and phenotypic defects were manually scored. Embryonic lethality was assessed by scoring unhatched embryos from 10×post-imaging whole well scans as previously described (10, 11). The feeding constructs for RNAi targeting *src-2* was selected from the Ahringer library, while the *src-1 (RNAi* ) feeding construct was kindly gifted by Dr. Erin Cram. The targeting insert of the *src-1 (RNAi)* feeding plasmid were amplified by two primers: F400 5' aggctagcgagagtgaagaatggtacg 3', and R1449 5' agagacagcattggcatacggtagcc (Cram et al., 2006). The *src-1 (RNAi)* feeding construct was used in the panel (D, F-H). RNAi bacteria were cultured until log phase was reached, and then spread onto MYOB plates supplemented with 1mM IPTG and 25 μg/ml carbenicillin. The seeded plates were incubated overnight before conducting the brood size assay. To knock down the expression of the target genes *src-1* and *src-2* , mid-L4 hermaphrodites were transferred onto the IPTG-induced RNAi plates. Animals were then grown on RNAi plates at 20°C for 60 hours for brood size and other assays. All DIC images were acquired using a spinning disk confocal system equipped with a Photometrics Prime 95B EMCCD camera, a Yokagawa CSU-X1 confocal scanner unit, and a Nikon 60X 1.2 NA water objective.

## Reagents

N2 : Bristol (wild type)

AG538 : *src-2 ( av233 ) [Val170Ile] I*

RB936 : *src-2 ( ok819 ) I*

AG605 : *src-2 ( av233 ) [Val170Ile] I ltSi249 [pOD1274/pSW098; P dlg-1 delta7:: dlg-1 -GFP:: unc-54 -3'UTR; cb-unc-119 (+)]I; ltSi511 [pOD2983/pSW207;P cnd-1 ::mCherry-PH:: unc-54 _3'UTR; cb-unc-119 (+)]II*

AG606 : *src-2 ( av233 ) [Val170Ile] I; stIs10389 [ pha-4 ::TGF(3E3)::GFP::TY1::3xFLAG inserted into fosmid WRM0617dE06 as C-terminal protein fusion]; ltSi539 [pOD1519/pSW224; P dlg-1 delta7::mCherry:: his-72 :: unc-54 _3’UTR; P cnd-1 ::mCherry:: his-72 :: unc-54 _3’UTR; cb-unc-119 (+)]II; ltSi507 [pOD1492/pSW201; Phlh1::GFP:: his-72 ::tbb-2_3’UTR, Phlh-1::mCherry:: his-72 ::tbb-2_3’UTR; cb-unc-119 (+)]IV*

AG757 *: src-1 ( av298 ) [Val190Ile] I*

AG625 : *src-1 ( av298 ) [Val190Ile] I src-2 ( av233 ) [Val170Ile] I*

AG607 : *src-1 ( av298 ) [Val190Ile] I src-2 ( av233 ) [Val170Ile] I; ltSi249 [pOD1274/pSW098; P dlg-1 delta7:: dlg-1 -GFP:: unc-54 -3'UTR; cb-unc-119 (+)]I; ltSi511 [pOD2983/pSW207;P cnd-1 ::mCherry-PH:: unc-54 _3'UTR; cb-unc-119 (+)]II*

OD1689 : *ltSi249 [pOD1274/pSW098; Pdlg1delta7:: dlg-1 -GFP:: unc-54 -3'UTR; cb-unc-119 (+)]I; ltSi511 [pOD2983/pSW207;P cnd-1 ::mCherry-PH:: unc-54 _3'UTR; cb-unc-119 (+)]II*

OD1719 : *stIs10389 [ pha-4 ::TGF(3E3)::GFP::TY1::3xFLAG inserted into fosmid WRM0617dE06 as C-terminal protein fusion]; ltSi539 [pOD1519/pSW224; P dlg-1 delta7::mCherry:: his-72 :: unc-54 _3’UTR; P cnd-1 ::mCherry:: his-72 :: unc-54 _3’UTR; cb-unc-119 (+)]II; ltSi507 [pOD1492/pSW201; Phlh-1::GFP:: his-72 ::tbb-2_3’UTR, Phlh-1::mCherry:: his-72 ::tbb-2_3’UTR; cb-unc-119 (+)]IV*

N2 and RB936 were obtained from the CGC.
